# Information transfer by local field potentials in the hippocampal formation

**DOI:** 10.1186/1471-2202-16-S1-P168

**Published:** 2015-12-18

**Authors:** Maria Constantinou, Daniel Squirrell, John Gigg, Marcelo A Montemurro

**Affiliations:** 1 Faculty of Life Sciences, University of Manchester, Manchester, M13 9PT, UK

## 

Extracellular electrical potential oscillations recorded as local field potentials (LFPs) in the hippocampal formation are thought to be involved in cognitive processes such as working memory retention, memory consolidation and spatial navigation. Recent studies have shown that combining spikes with LFP phase can increase the information content of spikes [1,2] and thus suggest LFP oscillations have the capacity to convey information. LFP oscillations within specific frequency bands can interact, for example by phase-phase and phase-amplitude coupling. We hypothesise that these LFP interactions can transfer information between neural networks. To test this hypothesis, we analyse multi-channel recordings of simultaneous LFPs from hippocampal area CA1 and the subiculum of urethane-anaesthetised rodents. Anatomical connections between these two regions in the hippocampal formation predict information in this system flows in 'nested loops' along separate projections [3]. We use advanced neurocomputational methods to determine how information flows within the CA1-subicular circuit by interactions of LFP rhythms. The results of correlation and coherence analyses of LFPs recorded from multiple sites suggest that LFP rhythms can transmit information within and between area CA1 and the subiculum. However, these methods alone are not enough to determine the oscillatory activity in which region drives activity in other regions, that is in which direction information flows. We use transfer entropy [4], which is an information theoretic method that can capture directionality, to quantify information transfer between area CA1 and subiculum. We show that information flow within the CA1-subicular circuit is bi-directional and follows a pattern of feedforward and feedback loops. Our results suggest that LFP interactions can route information at millisecond timescales along the anatomical connections in the hippocampal formation to achieve cognitive processing.
